# Cognitive Behavioral Therapy Versus Usual Care Before Bariatric Surgery: One-Year Follow-Up Results of a Randomized Controlled Trial

**DOI:** 10.1007/s11695-020-05081-3

**Published:** 2020-11-10

**Authors:** Linda Paul, Colin van der Heiden, Daphne van Hoeken, Mathijs Deen, Ashley Vlijm, René A. Klaassen, L. Ulas Biter, Hans W. Hoek

**Affiliations:** 1 PsyQ Department of Eating Disorders, Rotterdam, Netherlands; 2 Parnassia Psychiatric Institute, Kiwistraat 43, 2552 DH The Hague, Netherlands; 3 Institute of Psychology, Erasmus University, Rotterdam, Netherlands; 4 Institute of Psychology, Leiden University, Leiden, Netherlands; 5 Department of Surgery, Maasstad Hospital, Rotterdam, Netherlands; 6 Department of Surgery, Franciscus Gasthuis & Vlietland, Rotterdam, Netherlands; 7 Department of Psychiatry, University of Groningen, University Medical Center Groningen, Groningen, Netherlands; 8 Department of Epidemiology, Mailman School of Public Health, Columbia University, New York, NY USA

**Keywords:** Cognitive behavioral therapy, Bariatric surgery, Weight loss, Weight change, Obesity, Eating behavior, Eating disorders, Depression, Quality of life

## Abstract

**Abstract:**

**Background:**

Although early results of bariatric surgery are beneficial for most patients, some patients regain weight later. Cognitive behavioral therapy (CBT) has been suggested as a way to improve patients’ psychological health and maintaining weight loss in the longer term. The added value of preoperative CBT to bariatric surgery was examined. Pre- and posttreatment and 1-year follow-up data are presented.

**Methods:**

In a multi-center randomized controlled trial, CBT was compared to a treatment-as-usual (TAU) control group. Measurements were conducted pre- and posttreatment/pre-surgery (T0 and T1) and at 1-year post-surgery (T2). Patients in the intervention group received 10 individual, weekly sessions of preoperative CBT focused on modifying thoughts and behaviors regarding eating behavior, physical exercise, and postoperative life style. Outcome measures included weight change, eating behavior, eating disorders, depression, quality of life, and overall psychological health.

**Results:**

Though no significant differences between conditions were found per time point, in the CBT, condition scores on external eating, emotional eating, depressive symptoms, and psychological distress decreased significantly more over time between pre- (T0) and posttreatment (T1) pre-surgery compared to TAU. No significant time x condition differences were found at 1-year post-surgery (T2).

**Conclusions:**

Compared to TAU, preoperative CBT showed beneficial effects on eating behavior and psychological symptoms only from pretreatment to posttreatment/pre-surgery, but not from pre-surgery to 1-year post-surgery. Preoperative CBT does not seem to contribute to better long-term outcomes post-surgery. Recent studies suggest that the optimal time to initiate psychological treatment may be early in the postoperative period, before significant weight regain has occurred.

**Trial Registration:**

https://www.trialregister.nl Identifier: Trial NL3960.

## Introduction

Despite early positive outcomes, 20–30% of bariatric surgery patients experience suboptimal long-term results, including premature weight stabilization and weight regain in the years following surgery [1–5]. Weight change results after bariatric surgery show considerable individual differences, which might be partly related to psychopathology [2, 6, 7]. International prevalence data show that around 40% of bariatric surgery patients have at least one psychiatric diagnosis [8, 9]. Most common are depressive disorders, anxiety disorders, and eating disorders [9–12]. Studies investigating psychopathology using structured diagnostic interviews show preoperative prevalence rates of 31.5% for depression, up to 50% for disordered eating, including 5–15% for binge eating disorder, and up to 24% for anxiety disorders [13].

Research focused on predictors of (sustained) weight change after bariatric surgery has addressed both pre- and postoperative factors. Studies on the effect of preoperative patient characteristics such as the preoperative presence of psychiatric pathology (e.g., anxiety disorders) and of dysfunctional eating behaviors (e.g., grazing) and on the effect of preoperative preparation procedures (e.g., mandatory weight loss and lifestyle interventions) have yielded inconclusive evidence for the prediction of sustained postoperative weight change [14–20]. Postoperative factors appear to influence long-term weight change more directly than preoperative factors. Disordered eating, eating disorders, and depressive symptoms occurring in the postoperative phase, in particular, are associated with suboptimal weight change results and weight regain [21–27]. Further, problematic eating behaviors, such as binge eating and emotional eating, decrease initially after surgery but increase between 1 and 3 years postoperative [27]. As such, it may be necessary to provide postoperative interventions targeted at those who experience sub-optimal weight loss, weight regain, and/or the increase of problematic eating behaviors after surgery [22]. However, preoperative psychological interventions have the potential to prevent postoperative weight regain and promote psychological well-being.

Several studies have shown that cognitive behavioral therapy (CBT) is effective in reducing risk factors for weight regain, such as disordered eating behavior and depression [28–32]. CBT is based on the concept that thoughts, emotions, and behavior are interrelated. Treatment is focused on raising awareness of negative thoughts and emotions. CBT aims to help the patient to formulate more constructive thoughts and to make the desired behavior changes.

Gade et al. [28] investigated ten weekly sessions of CBT in bariatric surgery patients focused on improving eating behavior and mood and found that the CBT group showed a decrease in disordered eating and affective symptoms and a larger weight loss post intervention. Four years after surgery, this preoperative CBT intervention was associated with lower body weight as compared with TAU, but only in patients with minor or considerable symptoms of depression [29]. Telephone-based CBT (tele-CBT) was tested both pre- and postoperatively in two pilot studies [30, 31]. Results showed that six weekly sessions of tele-CBT focused on self-monitoring, problem-solving, and goal-setting improved eating behaviors and reduced mood and anxiety symptoms. Due to methodological shortcomings in these studies, such as small sample sizes and lack of control groups, the results cannot be generalized.

The current RCT aimed to investigate the added value of 10 sessions of CBT prior to bariatric surgery compared to the standard preparation/treatment-as-usual (TAU) procedure in the hospital for long-term maintenance of weight loss and psychological well-being [33]. For the completed 1-year follow-up, it was hypothesized that preoperative CBT aimed at improving eating behavior and mental health resulted in a greater reduction of maladaptive eating behavior, depressive symptoms and psychological distress, as well as increased QoL, as compared to the control group. Though 5% of the patients start to regain weight after 6 months post-surgery, average postoperative weight course shows that weight regain mainly occurs after the second year postoperative [34]. As such, differences in weight change were not expected to occur within the first year postoperative, but in the longer term.

## Materials and Methods

### Design and Setting

This study was conducted in a cooperation between PsyQ, a community mental health center that is part of the Parnassia Psychiatric Institute, and two general hospitals, all three located in Rotterdam, Netherlands. It was designed as a long-term multi-center RCT, to compare an intervention group given 10 weekly, individual sessions of preoperative CBT with a treatment-as-usual (TAU) control group given the regular preparation procedure for bariatric surgery in the hospitals.

Measurements have been carried out at three time points: pretreatment/pre-surgery (T0), posttreatment/pre-surgery (T1), and at 1-year post-surgery (T2).

### Sample Size and Randomization

To achieve a power of 0.80 (α = 0.05), 128 patients (64 patients per group) were needed. An online randomization list (www.randomization.com) was managed by an independent PsyQ office manager to ensure allocations were concealed.

### Patients

**Fig. 1:**
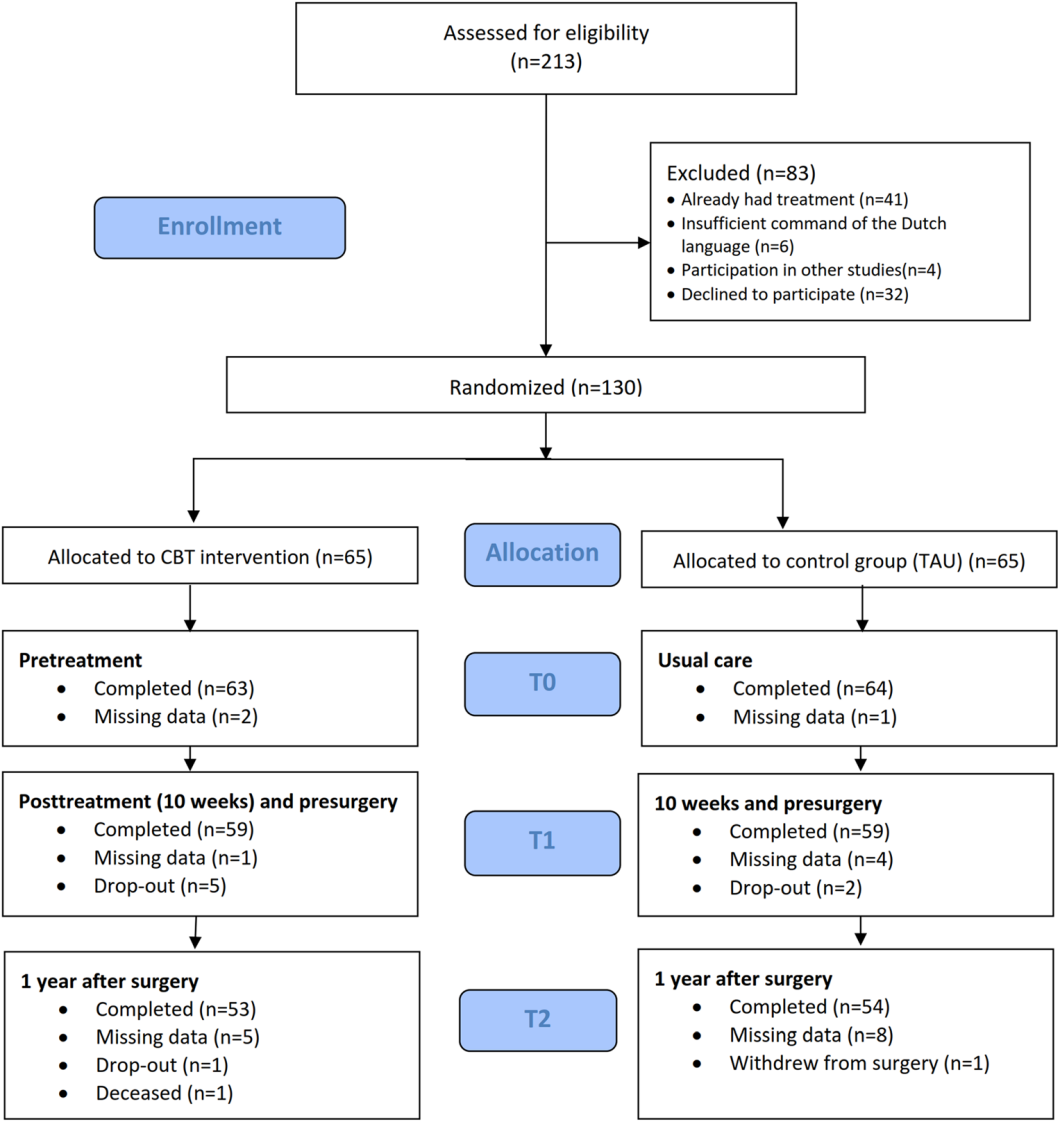
Participant flow through enrollment, allocation, and follow-up

Inclusion criteria were (1) patients who successfully passed preoperative screening and were on the waiting list for bariatric surgery in one of the hospitals and (2) aged between 21 and 65 years. Exclusion criteria were (1) current treatment by a dietitian, psychiatrist, or psychologist; (2) current psychotic or bipolar disorder, suicidality, or substance addiction; (3) poor command of the Dutch language; or (4) participation in another study on weight and bariatric surgery outcomes. In those cases where preoperative screening revealed a need for treatment of psychological or dietary problems, this type of treatment was offered. This treatment had to be completed prior to inclusion in the study. Those who had completed this psychological or dietary treatment were included and randomized over the treatment conditions of the study. Inclusion ran from January 2013 until July 2016. In total, 213 patients were assessed for eligibility at the bariatric surgery department of one of the hospitals. In total, 51 patients were excluded due to their current treatment (*n* = 41), poor command of Dutch (*n* = 6), or participation in another study (*n* = 4) (Fig. 1), and 32 patients declined to participate, so 130 patients were included for the study.

### Procedure

Preoperative screening for bariatric surgery included medical screening, up to three dietetic sessions to assess the nutritional status and the ability to change eating behavior, and standardized psychological assessment in an individual setting. In the psychological assessment the RAND-36 [35], a health-related quality of life measure investigating eight health concepts including emotional well-being and social functioning (the Dutch translation of a prequel of the SF36), the Dutch Eating Behavior Questionnaire (DEBQ) [36], a measure for disordered eating, and the SCL-90 [37], a checklist for mental and somatic complaints, were used. Patients who had passed this screening (*n* = 213) were invited to participate in the study. Two days after patients had been informed personally and in writing about the study, they were contacted by telephone. Those willing to participate and who met the inclusion criteria (130) were asked to provide written informed consent. They were then randomly assigned to either the CBT or the control group. Patients in the CBT group were scheduled for 10 weekly, individual face-to-face sessions of CBT at the eating disorders and obesity department of PsyQ, Rotterdam.

### Groups and Treatment

Patients in both groups followed the standard preoperative preparations of the hospital, consisting of a mandatory group meeting in which information on the surgery was provided. They were also given a booklet with detailed information on the surgery.

In addition to the regular preparations, patients in the intervention group were given 10 weekly, individual and face-to-face CBT sessions of 45-min duration. The objectives of the CBT intervention were to reduce dysfunctional eating behavior and improve mental health and lifestyle in terms of eating and activity, as well as preparation for the postoperative period. Patients needed to attend at least 6 CBT sessions to be considered a completer. In short, the protocol was based on CBT manuals developed for eating disorders [38] and obesity [39] and modified for bariatric surgery patients.

The CBT focused on nutritional and activity management (sessions 1–4), cognitive restructuring and developing alternative behavior (sessions 5–8), and relapse prevention strategies and preparation for the postoperative period (sessions 9–10) [33].

In the first session, information about obesity and the CBT intervention was provided, a weight graph was drawn to identify factors related to the course of weight (changes), and a cost-benefit analysis was made for lifestyle changes. Session two provided information about healthy food and eating habits. Nutritional management was discussed and an eating diary was introduced as a tool for self-monitoring of eating behavior. In session three, information about daily physical activity was provided and the history of physical activity was discussed. Keeping a physical activity diary was introduced as a tool to monitor healthy activity behavior and a week’s goal for eating was set, e.g., having breakfast daily. The fourth session focused on further lifestyle changes regarding eating and physical exercise. Also, mindful eating was practiced during the session.

In session five, cognitive restructuring was introduced by using the “ABC” model: (A) analyze activating event, (B) beliefs and thoughts, and (C) consequences—emotions and actions in problematic situations. Triggers for disordered eating were discussed, as well as alternative coping strategies such as expressing negative emotions and asking for support or planning mealtimes ahead. In session six, a behavioral analysis of an eating or physical activity situation with the ABC model was made, e.g., thoughts, emotions, and actions in a situation of overeating after a birthday party. Session seven focused on identifying obstructive thoughts and practicing replacing these with constructive thoughts. Session eight focused on further behavior change by reducing the risk of unplanned eating or overeating by developing alternative habits (e.g., eating from a smaller plate, using a shopping list) and adopting alternative behaviors for the urge to eat (e.g., taking a walk, listening to music).

In session nine, a relapse prevention plan was composed, bringing together (future) trigger situations and coping strategies to handle these situations. In session ten, the final relapse prevention plan was discussed as well as expectations of, and preparation for, surgery and the period thereafter. The intervention was also evaluated in this final session.

### Measures

Weight and height data were collected from the medical files. Clinical data regarding eating behavior, eating disorders, depression, QoL, and overall psychological health were collected using online questionnaires, prompted by automated emails with a link to fill in the questionnaires. All measures were collected or calculated at each time point.

#### Body Mass Index (BMI) and Weight Change

BMI was expressed in kg/m^2^. Weight change was defined as the percentage of total weight loss (%TWL). Weight was measured in the hospital through a standardized procedure on a calibrated scale by an impartial nurse or surgeon.

#### Eating Behavior

The Dutch Eating Behavior Questionnaire (DEBQ) was used to assess eating behavior [36, 40]. The DEBQ distinguishes three subscales: emotional eating, external eating, and restrained eating. The 33 items can be answered on a 5-point Likert scale ranging from 1 “never” to 5 “very often.” The three DEBQ scales have good psychometrical qualities [41].

#### Eating Disorders

Eating disorder symptomatology was measured by the Eating Disorder Examination Questionnaire (EDE-Q) [42], Dutch translation by Nauta [43]). This self-report questionnaire comprises 22 items in four subscales, which can be answered on a 7-point Likert scale ranging from 0 “not one day” to 6”‘every day.” Each subscale score is calculated from the sum and average of its items. The global EDE-Q score is calculated by summing and averaging all four subscale scores. The EDE-Q has adequate internal consistency and test-retest reliability [44, 45]. Its validity has not been well established, but two studies have shown accurate discriminative validity [46, 47].

#### Depressive Symptoms

The Quick Inventory of Depressive Symptomatology-Self-Rating (QIDS-SR) [48] is a self-report questionnaire for measuring depressive symptomatology related to the nine symptom domains of DSM-IV’s major depressive disorder criteria. There are 16 items to answer on a 4-point Likert scale ranging in severity from 0 “low” to 3 “high.” The sum of the scores for each of the symptom domains determines the total score. The QIDS-SR has high internal consistency and high concurrent validity [48].

#### Quality of Life

The short version of the World Health Organization Quality of Life (WHOQoL-BREF) [49] is a self-report questionnaire with 26 items to assess QoL. It measures four domains: physical health, psychological health, social relationships, and environment. Answers are given on a 5-point Likert scale ranging from 1 “very dissatisfied” to 5 “very satisfied.” The mean scores of items in each domain are used to calculate the domain scores. WHOQoL-BREF domain scores demonstrate good discriminative validity, content validity, internal consistency, and test-retest reliability [50].

#### Psychological Distress

The Brief Symptom Inventory (BSI) [51, 52] was used to assess the level of psychological distress. This self-report questionnaire has 53 items, which are ranked on a 5-point Likert scale ranging from 0 “not at all” to 4 “extremely.” The Global Severity Index (GSI) was used to indicate the overall level of psychological distress. The instrument has good internal reliability, convergent validity, and test-retest reliability [53, 54].

### Supervision and Fidelity Monitoring

Seven certified CBT therapists were trained to use the treatment manual. Additionally, each therapist observed all the CBT sessions of a bariatric surgery patient treated by the first author (LP). During the study, there were monthly supervision meetings with the first (LP) and second (CH) authors, in which all active cases and therapy notes were reviewed against an intervention checklist to ensure adherence to the manual and the quality of treatment.

### Ethics

The study was approved by the Medical Research Ethics Committees United (MEC-U) and registered in the Dutch Trial Register, http://www.trialregister.nl (ID no. NTR4140).

### Statistical Analysis

Continuous variables were described using means and standard deviations, categorical data as counts and percentages. For continuous data, *t*-tests were used to assess group differences, and Benjamini-Hochberg correction [55] was used to correct for multiple testing. Clinical outcome measures were at interval or ratio level. Therefore, linear mixed models (LMM) with a random intercept were used. Outcomes during treatment (T0-T1) and post-surgery (T1-T2) were analyzed separately. LMM predictors included group (control vs. treatment) and time (T0-T1 and T1-T2). In the analyses regarding post-surgery outcomes, the baseline score (T0) of the outcome was controlled for. The interaction between condition and time was included in the models if this would lead to an improved model fit, as assessed by a likelihood ratio test. The analyses were carried out according to the intention-to-treat strategy [56] using SPSS-25.

## Results

### Recruitment and Patient Flow

In total, 130 patients were randomly allocated to the CBT intervention or control group (see Fig. 1). During the course of the study, eight patients dropped out (four due to personal circumstances, one because of logistical problems, and three declined further participation, and did not complete the minimal number of 6 sessions). In addition, one patient died in the year after surgery and another one declined surgery.

Data on weight were complete for 99% of participants at T0, 100% at T1, and 100% at T2 (including one self-report). Questionnaire data were complete for 98% at T0, 91% at T1, and 82% at T2.

### Sample Description

Descriptive data on the sample are presented in Table 1. Most patients were female (74%), married (65%), had finished middle education level (65%), and were employed part- or full-time (74%). The age difference between the randomized CBT and control groups was found to be statistically significant (*t*(120.23) = 2.90, *p* < 0.01, *d* = 0.51).

**Table 1:** Baseline demographic characteristics^a^

	Total (*N* = 128)		CBT (*n* = 63)		Control (*n* = 65)		*p*^b^
Age	41.7	(9.7)	44.1	(8.2)	39.3	(10.6)	< 0.01
Female	95	(74)	46	(73)	49	(75)	0.84
Living conditions							0.14
Married or cohabitating	83	(65)	45	(71)	38	(58)	
Single or divorced	45	(35)	18	(29)	27	(42)	
Education level							0.85
High	34	(27)	18	(29)	16	(25)	
Middle	83	(65)	40	(64)	43	(66)	
Low	11	(9)	5	(8)	6	(9)	
Employed	95	(74)	47	(75)	48	(74)	>0.999

^a^Data presented as observed mean (SD) or number (%)

^b^Significance values are based on independent samples *t-*tests for continuous variables and on Fisher’s exact test for categorical variables

### Outcomes

Observed mean scores for clinical outcome measures per group and time point are shown in Table 2. At T2, BMI scores (*t*(119) = − 1.04, *p* = 0.69, *d* = − 0.19) did not show significant differences between the CBT and the TAU conditions. At the various time points, no significant differences between the two conditions were found for the outcome measures eating behavior, eating disorders, depression, quality of life, and overall psychological health.

**Table 2:** Observed means (SD) per time point and treatment condition

	T0				T1				T2			
	CBT	control	*t*(*df*)	*p*	CBT	control	*t*(*df*)	*p*	CBT	control	*t*(*df*)	*p*
BMI	42.7 (5.0)	43.4 (5.4)	− 0.76 (123)	0.82	42.1 (4.9)	43.2 (5.8)	− 1.11 (121)	0.67	29.2 (4.6)	30.1 (4.7)	− 1.04 (119)	0.69
% TWL	–	–	–	–	1.0 (3.2)	0.5 (3.8)	0.75 (121)	0.82	31.4 (7.7)	30.7 (7.3)	0.51 (119)	0.86
DEBQ external eating	28.4 (6.0)	27.3 (5.7)	1.14 (123)	0.67	25.7 (5.4)	27.3 (5.4)	− 1.56 (117)	0.49	23.0 (5.2)	23.4 (5.2)	− 0.45 (105)	0.86
DEBQ restrained eating	32.4 (6.1)	31.0 (6.2)	1.27 (123)	0.63	30.2 (5.7)	30.1 (6.3)	0.12 (117)	0.93	29.5 (6.2)	30.0 (6.2)	− 0.45 (105)	0.86
DEBQ emotional eating	31.6 (10.4)	26.5 (8.3)	3.09 (123)	0.12	28.0 (9.9)	26.5 (9.3)	0.88 (117)	0.74	25.9 (9.6)	23.6 (9.4)	1.3 (105)	0.63
EDEQ total score	2.5 (0.9)	2.4 (0.9)	0.50 (125)	0.86	2.0 (0.8)	2.0 (0.9)	0.16 (117)	0.93	1.3 (0.9)	1.5 (1.0)	1.58 (108)	0.82
QIDS total score	6.3 (4.4)	4.8 (3.0)	2.23 (111.7)	0.22	4.9 (3.4)	4.7 (3.4)	0.32 (117)	0.90	4.6 (3.7)	4.6 (3.3)	0.04 (107)	0.97
WHOQoL physical health	12.5 (2.9)	13.3 (2.8)	− 1.66 (124)	0.49	12.8 (3.0)	13.7 (2.9)	−1.63 (117)	0.49	15.1 (2.8)	15.3 (3.0)	− 0.38 (107)	0.86
WHOQoL psychological health	13.4 (2.4)	14.6 (2.0)	− 2.95 (124)	0.12	13.8 (2.1)	14.7 (2.5)	− 2.17 (117)	0.22	15.0 (2.3)	15.2 (2.7)	− 0.42 (107)	0.86
WHOQoL social relations	13.5 (2.3)	14.6 (2.4)	−2.62 (124)	0.12	13.6 (2.6)	14.3 (2.9)	−1.37 (117)	0.63	14.3 (2.8)	14.5 (3.7)	−0.20 (107)	0.93
WHOQoL environment	15.0 (1.9)	15.4 (2.3)	− 1.00 (124)	0.70	15.2 (2.0)	15.4 (2.6)	− 0.50 (117)	0.86	15.6 (2.2)	15.5 (3.1)	0.24 (107)	0.93
BSI GSI score	1.5 (0.6)	1.3 (0.4)	2.00 (98.9)	0.29	1.4 (0.3)	1.5 (0.6)	− 1.22 (86.6)	0.63	1.4 (0.6)	1.5 (0.5)	− 0.18 (97)	0.93

Data are presented as observed mean (SD). Benjamini-Hochberg correction was used to correct for multiple testing

Time points: T0 pretreatment/pre-surgery; T1 posttreatment/pre-surgery; T2 1-year post-surgery

Treatment groups: CBT = treatment as usual + cognitive behavior therapy; controls had treatment as usual

*BMI* body mass index, *BSI* Brief Symptom Inventory, *DEBQ* Dutch Eating Behavior Questionnaire, *EDE-Q* Eating Disorder Examination Questionnaire, *GSI* Global Severity Index, *QIDS* Quick Inventory of Depressive Symptomatology, *TWL* total weight loss, *WHOQoL* World Health Organization Quality of Life

The results of the analyses of treatment condition and time effects are shown in Table 3. Comparing pre- to posttreatment data (T0-T1), for both groups time proved to be the most important predictor for the observed improvement in restrained eating and eating disorder pathology. For external eating, emotional eating, depressive symptoms, and psychological distress, the time x condition model was superior: scores decreased significantly over time for the CBT group only. For the QoL scales, there were no statistically significant main effects for time, nor was a model with an interaction effect superior.

**Table 3:** Parameter estimates for mixed models regarding T0 and T1 and T1 and T2 corrected for T0

	T0-T1			T1-T2 | T0		
	Estimate	SE	*p*	Estimate	SE	*p*
DEBQ external						
Intercept	**27.18**	**0.70**	**< 0.01**	**13.26**	**1.49**	**< 0.01**
Condition	1.19	0.99	0.23	**− 1.48**	**0.56**	**< 0.01**
Time	0.25	0.51	0.62	**− 3.30**	**0.45**	**< 0.01**
Baseline	NA	NA	NA	**0.63**	**0.05**	**< 0.01**
Time x condition	**− 2.62**	**0.72**	**< 0.01**			
DEBQ restrained						
Intercept	**31.50**	**0.72**	**< 0.01**	**17.37**	**2.35**	**< 0.01**
Condition	0.77	0.97	0.43	− 0.72	0.81	0.38
Time	**− 1.92**	**0.50**	**< 0.01**	− 0.50	0.63	0.43
Baseline	NA	NA	NA	**0.42**	**0.07**	**< 0.01**
Time x condition						
DEBQ emotional						
Intercept	**26.39**	**1.19**	**< 0.01**	**11.24**	**2.08**	**< 0.01**
Condition	**5.14**	**1.69**	**< 0.01**	− 0.76	1.09	0.49
Time	0.18	0.98	0.86	**− 2.78**	**0.86**	**< 0.01**
Baseline	NA	NA	NA	**0.67**	**0.06**	**< 0.01**
Time x condition	**− 3.23**	**1.39**	**0.02**			
EDE-Q						
Intercept	**2.43**	**0.10**	**< 0.01**	**1.49**	**0.22**	**< 0.01**
Condition	0.06	0.14	0.68	− 0.05	0.11	0.63
Time	**− 0.43**	**0.07**	**< 0.01**	**− 0.64**	**0.10**	**< 0.01**
Baseline	NA	NA	NA	**0.49**	**0.06**	**< 0.01**
Time x condition						
QIDS						
Intercept	**4.79**	**0.45**	**< 0.01**	**2.68**	**0.62**	**< 0.01**
Condition	**1.51**	**0.64**	**0.02**	− 0.70	0.45	0.12
Time	− 0.03	0.40	0.93	− 0.38	0.31	0.23
Baseline	NA	NA	NA	**0.53**	**0.06**	**< 0.01**
Time x condition	**− 1.26**	**0.57**	**0.03**			
BSI						
Intercept	**1.35**	**0.06**	**< 0.01**	**0.70**	**0.15**	**< 0.01**
Condition	0.16	0.09	0.06	− 0.12	0.07	0.07
Time	0.10	0.07	0.15	0.07	0.06	0.27
Baseline	NA	NA	NA	**0.48**	**0.08**	**< 0.01**
Time x condition	**− 0.26**	**0.10**	**< 0.01**			
Weight						
Intercept	**128.38**	**2.78**	**< 0.01**	**57.51**	**3.51**	**< 0.01**
Condition	− 2.18	3.94	0.58	− 0.78	1.04	0.46
Time	**− 0.89**	**0.40**	**0.03**	**− 38.53**	**1.04**	**< 0.01**
Baseline	NA	NA	NA	**0.85**	**0.02**	**< 0.01**
Time x condition						
WHOQoL physical health						
Intercept	**13.37**	**0.34**	**< 0.01**	**3.00**	**0.92**	**< 0.01**
Condition	− 0.86	0.47	0.07	− 0.13	0.34	0.71
Time	0.30	0.19	0.12	**2.08**	**0.26**	**< 0.01**
Baseline	NA	NA	NA	**0.64**	**0.06**	**< 0.01**
Time x condition						
WHOQoL psychological health						
Intercept	**14.59**	**0.27**	**< 0.01**	**3.49**	**1.01**	**< 0.01**
Condition	**− 1.11**	**0.38**	**< 0.01**	0.25	0.30	0.39
Time	0.17	0.16	0.29	**0.90**	**0.20**	**< 0.01**
Baseline	NA	NA	NA	**0.69**	**0.06**	**< 0.01**
Time x condition						
WHOQoL social relations						
Intercept	**14.51**	**0.30**	**< 0.01**	**3.86**	**1.35**	**<0.01**
Condition	**−0.93**	**0.41**	**0.02**	0.35	0.41	0.39
Time	−0.16	0.21	0.45	0.51	0.29	0.08
Baseline	NA	NA	NA	**0.66**	**0.08**	**<0.01**
Time x condition						
WHOQoL environment						
Intercept	**15.35**	**0.27**	**< 0.01**	2.06	1.08	0.06
Condition	−0.32	0.37	0.39	0.25	0.28	0.38
Time	0.02	0.15	0.90	**0.35**	**0.18**	**0.05**
Baseline	NA	NA	NA	**0.83**	**0.07**	**<0.01**
Time x condition						

Time x condition is available for outcomes where the interaction was in the selected model

NA = Not Applicable; bold face: *p* < 0.05

Comparing posttreatment to 1-year follow-up data, controlled for pretreatment data (T1-T2 | T0), the baseline covariates were significant in all the models. Main effects for time, indicating improvement for both groups, were found for external eating, emotional eating, eating disorder pathology, QoL physical health, and QoL psychological health. For none of the outcomes, the model with a time x condition interaction was superior.

## Discussion

In this large RCT, the effect of preoperative CBT compared to TAU was investigated in bariatric surgery patients posttreatment/pre-surgery and at 1-year post-surgery. Results showed an initial decrease in scores from pretreatment/pre-surgery to posttreatment/pre-surgery (T0-T1) in the CBT group regarding external eating, emotional eating, depressive symptoms, and psychological distress compared to the TAU group. However, at 1-year post-surgery, there were no differences between the CBT and the TAU groups on weight change, disordered eating, eating disorders, depression, quality of life, and overall psychological health.

These results are in line with the Norwegian RCT which ran simultaneously to this study [28] and which found pre-surgery improvement in disordered eating and affective symptoms in the CBT group, but no differences between CBT and TAU at 1-year post-surgery, and with Kalarchian et al. [15] who found that preoperative lifestyle intervention did not improve weight change at 24 months post-surgery. The three RCTs conducted so far on the effect of preoperative psychological interventions, including the current RCT, have not shown evidence that pre-surgery interventions improve weight loss and mental health following bariatric surgery beyond the effect of surgery in the longer term.

These results add to the findings of a systematic review of pre- and postoperative psychosocial interventions for bariatric surgery patients [57]. The authors report strongest evidence for the impact of psychosocial interventions, CBT in particular, on eating behaviors and psychological functioning. Psychological interventions focused on weight loss, dietary behaviors, and lifestyle behaviors show relatively weak and mixed results. They also conclude that the optimal time to initiate this type of treatment appears to be early in the postoperative period, before significant weight regain has occurred. Conceiçao et al. [22] argue that a stepped-care approach in the postoperative period may improve weight outcomes in a cost-effective way.

The current RCT adds to the accumulated body of evidence that preoperative CBT has an effect on eating behavior and psychological functioning posttreatment/pre-surgery, but that this effect does not last during the postoperative phase. No support was found for the hypothesis that preoperative CBT improves mental health and prevents weight regain in the postoperative phase.

The strengths of this study include its randomized design in a multi-center context. The research sample of bariatric surgery patients was large, using broad inclusion criteria. Commitment of patients to the CBT intervention was high with a mean number of 8.5 out of 10 completed sessions and with high initial and follow-up response rates for both groups.

One limitation was the lack of an active control condition. Another limitation was the administering of the intervention to all patients instead of a subset. The cost-effectiveness of additional (pre- or post-surgery) interventions to improve long-term results of bariatric surgery—such as preoperative CBT—would be improved when these could be targeted towards the 20–30% of bariatric surgery patients who experience suboptimal results from surgery. The current study was designed in 2012, and available knowledge at that time did not indicate specific preoperative risk factors for postoperative weight change. Even to date, no indicators are available that have sufficient sensitivity and specificity to screen bariatric surgery candidates and target additional interventions to only a subset. It was only in 2018 when the first RCT provided evidence that for patients with minor or considerable symptoms of depression CBT contributed to more weight change 4 years post-surgery [29]. This is a promising, but still tentative, avenue which is as yet too premature to use as screening criterion. The ongoing 3- and 5-year follow-ups of the current study may add relevant knowledge on this topic.

Another limitation of the study was that, despite randomization, there was a significant age difference between the two groups. To check whether this would interfere with the results, correlations between age, clinical outcomes at T0, and T0-T2 difference scores were investigated. There was one single significant, but weak (*r* = 0.19, *p* = 0.03) correlation between age and emotional eating at T0, which disappeared after correction for multiple testing (Bonferroni *p* = 0.85). Thus, it was not expected that age differences affected the results.

Future studies investigating CBT in bariatric surgery patients should focus on which clinical symptoms (e.g., mood or disordered eating behavior) and which subgroups of patients the interventions should be targeted, as well as on the long-term results on weight and psychological well-being.

## Conclusions

Compared to TAU, preoperative CBT showed beneficial effects on eating behavior and psychological symptoms only from pretreatment to posttreatment/pre-surgery, but not from pre-surgery to 1 year post-surgery. Preoperative CBT does not seem to contribute to better long-term outcomes post-surgery. Recent studies suggest that the optimal time to initiate psychological treatment may be early in the postoperative period, before significant weight regain has occurred.
